# Increased tuberculosis case detection in Tanzanian children and adults using African giant pouched rats

**DOI:** 10.1186/s12879-024-09313-0

**Published:** 2024-04-15

**Authors:** Tefera B. Agizew, Joseph Soka, Cynthia D. Fast, Stephen Mwimanzi, Gilbert Mwesiga, Nashon Edward, Marygiven Stephen, Reheme Kondo, Robert Burny, Christophe Cox, Negussie Beyene

**Affiliations:** 1 Anti-Persoonsmijnen Ontmijnende Product Ontwikkeling (APOPO) Tuberculosis Department, Sokoine University of Agriculture, Morogoro, Tanzania; 2 Department of Biology, University of Antwerp, Antwerp, Belgium; 3 APOPO, TB Detection Program, Eduardo Mondlane University, Maputo, Mozambique; 4 APOPO TB Research Project, Armauer Hansen Research Institute, Addis Ababa, Ethiopia

**Keywords:** Rat-based TB detection, Mycobacterium bacillary load, Pulmonary TB, APOPO

## Abstract

**Background:**

African giant pouched rats, trained by Anti-Persoonsmijnen Ontmijnende Product Ontwikkeling (APOPO), have demonstrated their ability to detect tuberculosis (TB) from sputum. We assessed rat-based case detection and compared the *mycobacterium* bacillary load (MTB-load) in children versus adults.

**Methods:**

From January–December 2022, samples were collected prospectively from 69 Directly Observed Therapy (DOT) facilities’ presumed TB patients. Using an average of five rats, APOPO re-evaluated patients with bacteriologically negative (sputum-smear microscopy or Xpert MTB/RIF) results. Rat-positive samples were tested using concentrated smear light-emitting diode microscopy to confirm TB detection before treatment initiation. The rats’ identification of pulmonary TB is based on smelling TB-specific volatile organic compounds (VOCs) in sputum. Using STATA, Chi-square for odds ratio and confidence interval was calculated and evaluated: (1) the yield of rat-based TB detection compared to that of the health facilities; (2) rat-based TB detection in children versus adults; and (3) rats’ ability to detect TB across MTB-loads and between children and adults.

**Results:**

From 35,766 patients, 5.3% (1900/35,766) were smear-positive and 94.7% (33,866/35,766) were smear or Xpert-negatives at DOTS facility. Of those with negative results, 2029 TB cases were detected using rats, contributing to 52% (2029/3929 of total TB identified), which otherwise would have been missed. Compared to DOT facilities, rats were six-fold more likely to detect TB among Acid Fast Bacilli (AFB) 1+/scanty [90% (1829/2029) versus 60% (1139/1900), odds ratio, OR = 6.11, 95% confidence interval, CI: 5.14–7.26]; twice more likely to identify TB cases among children [71% (91/129) versus 51% (1795/3542), OR = 2.3, 95% CI: 1.59–3.42]; and twice more likely to identify TB cases among children with AFB 1+/scanty than adults with the same MTB-load [5% (86/1703) versus 3% (28/1067), OR = 2.0, 95% CI: 1.28–3.03].

**Conclusions:**

Rats contributed over half of the TB cases identified in program settings, and children, especially those with a lower MTB-load, were more likely to be diagnosed with TB by rats. The chemical signatures, VOCs, were only available for adults, and further research describing the characteristics of VOCs in children versus adults may pave the way to enhance TB diagnosis in children.

## Introduction

Despite the curable and preventable nature of tuberculosis (TB), about 10.6 million people fell ill with it and 1.6 million died in 2021 [1]. The African region accounts for nearly one-third of the estimated global burden of TB, and TB remains a public health problem in sub-Saharan African countries, Tanzania included. TB is declining by only 5% on average annually in Tanzania [1]. With this current rate of decline, considerable gaps persist in finding and treating TB cases timely, as a result of which achieving reduced TB transmission and TB cases per the END TB target may not be possible in the near future [2]. Therefore, scaling up of cheaper, faster, and more sustainable diagnostic tests to find more TB patients, especially those missed in routine program settings, is essential.

Over the years, African giant pouched rats (*Cricetomys ansorgei*), trained by Anti-Persoonsmijnen Ontmijnende Product Ontwikkeling (APOPO) to identify TB by smell, have demonstrated their ability to detect TB from sputum [3–5]. The trained rats have very high sample throughput, as they can screen 100 sputum samples in 20 minutes [6]. The rats’ identification of pulmonary TB is based on smelling TB-specific volatile organic compounds (VOCs) in sputum specimens [5]. By the end of 2022, APOPO’s TB Detection Programs had screened 870,777 sputum samples from 517,264 presumptive TB patients and found 26,084 newly diagnosed TB patients in three high-TB burden countries (Ethiopia, Mozambique, and Tanzania). At APOPO, we have generally done this by re-evaluating (second-line TB screening) sputum samples initially tested and declared bacteriologically negative by sputum-smear microscopy (smear) or Xpert MTB/RIF [4]. Before treatment initiation, all TB patients identified using rats are confirmed using the national standard diagnostics.

Due to the low bacillary load and lesser sensitivity of the available diagnostic methods, including culture, diagnosing TB in children is difficult, leaving them undiagnosed and therefore untreated [7]. Using APOPO rats, Mgode et al. reported on the diagnosis of TB in children (0–14 years) in 2018. They discovered 39% (208/539) contributions among pediatric TB cases reported, which translates to 62.8% additional cases (incremental yield) by rats to DOT facilities TB case detection [8]. There is limited data on rats’ abilities to detect TB in children versus adults. Other than the above report, all the previous studies were focused on adults [9, 10]. In this study, we aim to evaluate: (1) the yield of rat-based TB case detection compared to that of the health facilities; (2) rat-based TB case detection in children versus adults; and (3) rats’ ability to detect TB across *Mycobacterium* TB bacillary loads (MTB-load) and between children and adults.

## Methods

### Study settings and designs

We conducted a prospective study using routinely collected data on presumptive TB patients enrolled in the national TB program’s routine care at 69 (58 Dar es Salaam, 11 Dodoma) DOT facilities from 1st of January to 31st of December 2022 in Tanzania.

### Study population

The study population includes all individuals of all ages, including children, men, and women, who presented with symptoms of TB at study sites.

### Tuberculosis screening

At the health facility visit, children (0–14 years old) and adults (15 years and above, hereafter referred to as adults) were screened for TB symptoms. Per the national TB guidelines, adults were screened for 4 TB symptoms (cough, fever, night sweats, and weight loss) of two or more weeks duration. Children were screened for weight loss or failure to thrive (no weight gain > 3 months), cough for ≥2 weeks, fever for ≥2 weeks, fatigue or reduced playfulness for ≥2 weeks, and profuse night sweats for ≥2 weeks [11]. Presumptive TB cases were defined when patients were screened positive for one or more of the TB symptoms using the above guide.

### APOPO tuberculosis detection model using rats

**Fig. 1:**
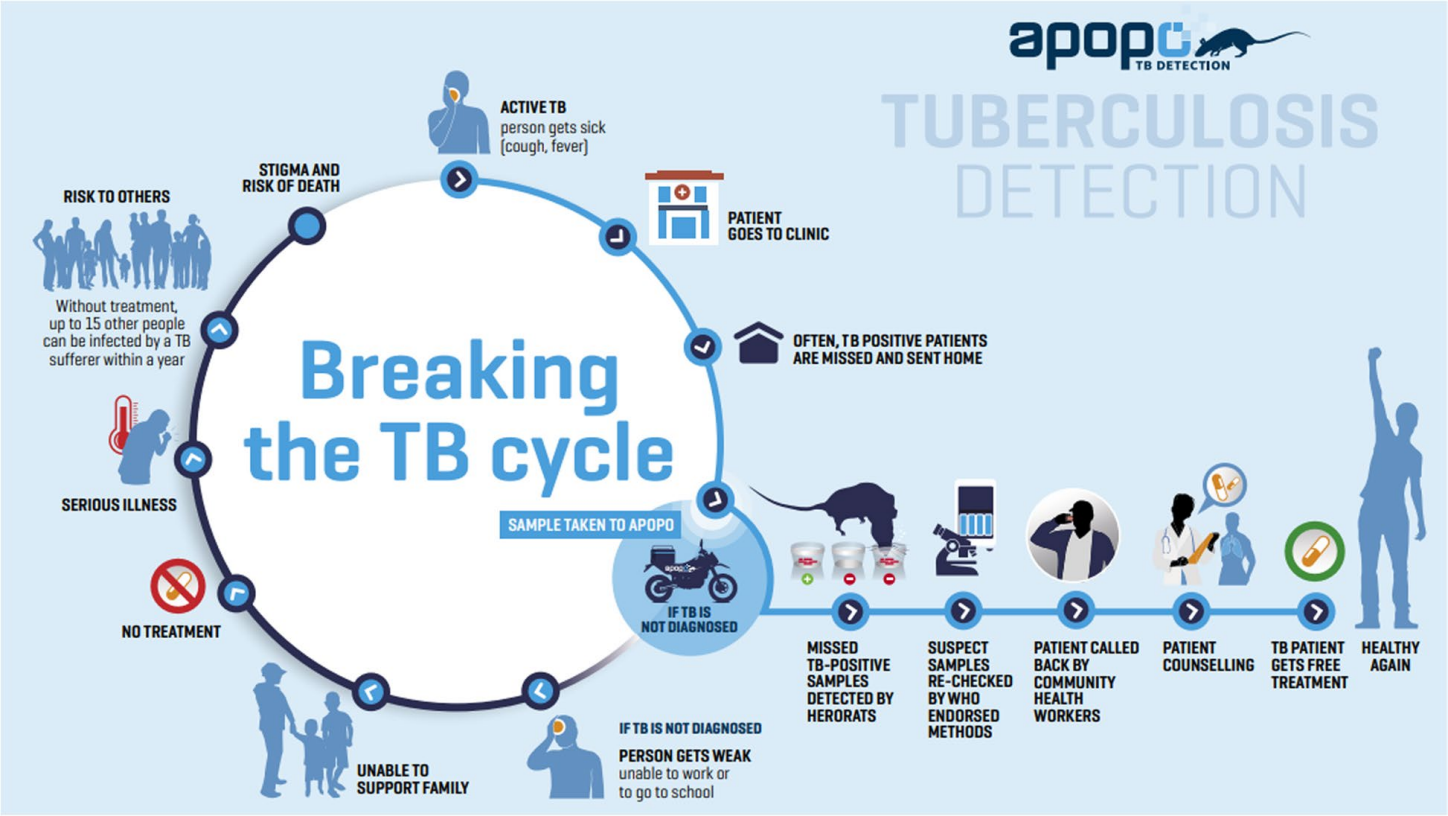
APOPO tuberculosis detection model using rats. Reproduced with permission [12]

APOPO sample collection, a rat-based assessment procedure, and a TB detection model using rats were reported in previous APOPO publications (Fig. 1) [3, 12]. In summary, smear, which has very good specificity but very poor sensitivity, especially in sub-Saharan African settings [13], remains the most commonly used TB diagnostic method in low- and middle-income countries [4]. At APOPO, samples from smear- or Xpert MTB/RIF-negative presumptive TB patients are screened using detection rats, and rat-positive samples are confirmed using light-emitting diode fluorescence smear microscopy (LED-FM), yielding an annual average of a 40% increase (incremental yield) in smear-positive case detection [3]. Therefore, APOPO envisages targeting and reaching the missed TB cases to maximize TB case detection and treatment coverage. In this study, five rats on average sequentially evaluated the samples placed under 10 sniffing holes in the floor in a of rectangular chamber (205 cm long, 55 cm wide, and 55 cm high). The details of the evaluation setup have been reported elsewhere [14].

### Data collection

Data were collected using standardized case report forms (CRF) between January and December 2022. An APOPO-developed TB Laboratory Information System (TB-LIMS) was used to record the data. Logic checks were used to find inconsistencies, which were then verified against the original CRF after being discovered. Consistencies and missing data were, whenever possible, fixed by reviewing patient charts.

### Statistical analysis

Data were analyzed using STATA statistical software 15 [15]. A Chi-square for odds ratio was calculated to analyze the demographic and laboratory parameters of the patients, and rats’ TB detection over the DOT facilities and compared the increase by MTB-load in children versus adults. We employed an odds ratio and a 95% confidence interval, and statistical significance was determined by *P values* less than 0.05.

## Results

**Fig. 2:**
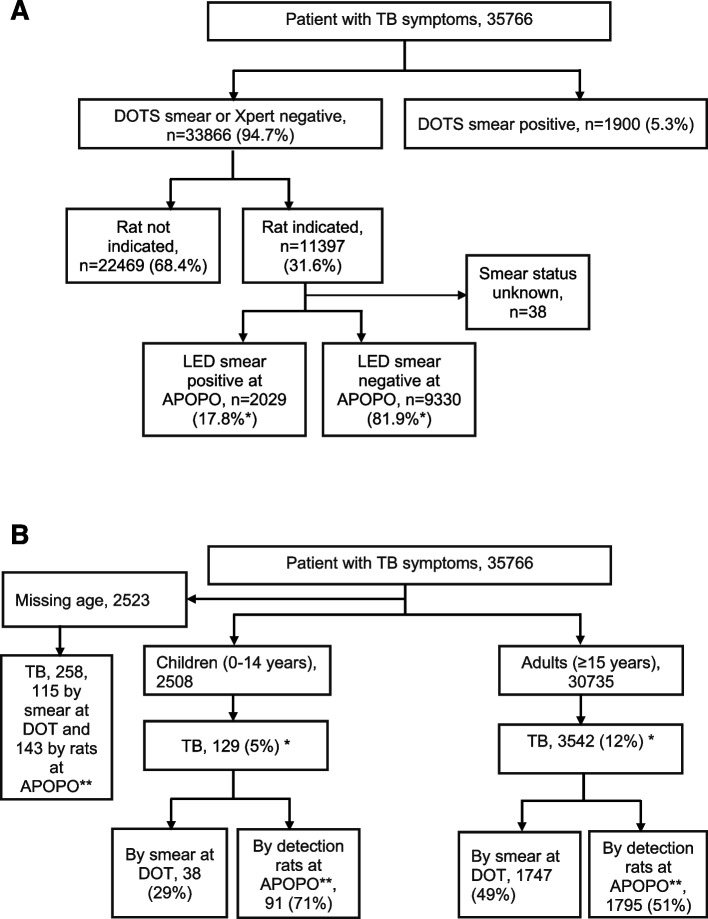
**A** Rat indication and tuberculosis cases after LED smear confirmation among presumptive tuberculosis patients in Tanzania study sites from January to December 2022. Note: *Since the samples were not tested by culture, 17.8% does not truly represent sensitivity. Since the LED smear is not good enough in sensitivity, nor does 81.9% represent a true false positive. **B** Presumptive tuberculosis and bacteriologically confirmed tuberculosis cases in Tanzania study sites from January to December 2022. Note: *Children versus adults bacteriologically confirmed TB: 5% (129/2508) versus 12% (3542/30,735), odds ratio 0.42, 95% confidence interval: 0.35–0.50, *p* value, 0.001

From a total of 35,766 patients, 46,048 samples were screened between January and December 2022; Among these patients 5.3% (1900/35,766) were smear-positive and 94.7% (33,866/35,766) were smear or Xpert-negatives at DOTS facility (Fig. 2a). Figure 2b shows children and adults, respectively, were 8% (2508/33,243) and 92% (30,735/33,243). Among presumptive TB patients 2523, and 258 TB patients were not included for the odds ratio analysis due to missing age. Bacteriologically confirmed (smear at DOT facilities and rats’ detection verified by smear) cumulative TB cases were 11% (3929/35,766), whereas 5% (129/2508) and 12% (3542/30,735) were among children and adults, respectively. The median age was 36 years (interquartile range, 26–47 years), and 68% (2680/3903) were males (Fig. 2b and Table 1).

**Table 1:** Characteristics of tuberculosis patients in Tanzania evaluated using rats at APOPO and sputum-smear microscopy at DOT facilities

Characteristic		TB by detection rats at APOPO^a^	TB by smear at DOT lab^**b**^			
	N	n (%)	n (%)	OR	95% CI	***p-***value
Age						
< 15 years	129	91 (71)	38 (29)	2.33	1.59–3.41	< 0.001
≥ 15 years	3542	1795 (51)	1747 (49)			
Gender						
male	2680	1281 (48)	1399 (52)	0.58	0.51–0.67	< 0.001
female	1224	748 (61)	476 (39)			
Bacillary load (AFB)						
scanty	2046	1466 (72)	580 (28)	6.11^c^	5.14–7.26	< 0.001
1+	922	363 (39)	559 (61)			
2+	549	148 (27)	401 (73)			
3+	412	52 (13)	360 (87)			

^a^TB at APOPO lab were Acid Fast Bacilli (AFB) negative at DOT facilities, then re-evaluated by rats and confirmed later using concentrated smear light-emitting diode microscopy. ^b^TB at DOT facilities were evaluated by sputum-smear microscopy and were positive for Acid Fast Bacilli. ^c^The Odds ratio was calculated by combining scanty and AFB 1+ together and AFB 2+ and 3+ together

*APOPO* Anti-Persoonsmijnen Ontmijnende Product Ontwikkeling

*DOT* Directly Observed Therapy

### TB detection using rats

**Fig. 3:**
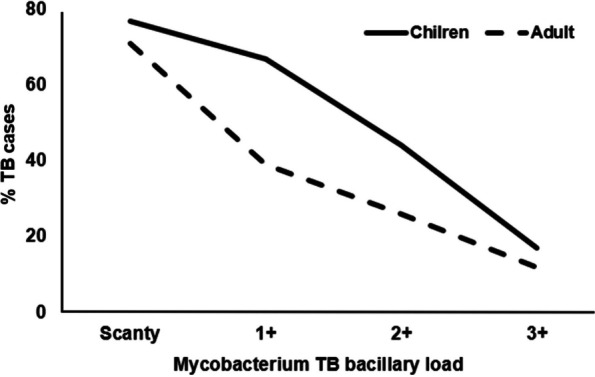
Tuberculosis cases among children and adults identified using rats by *Mycobacterium* bacillary load

**Fig. 4:**
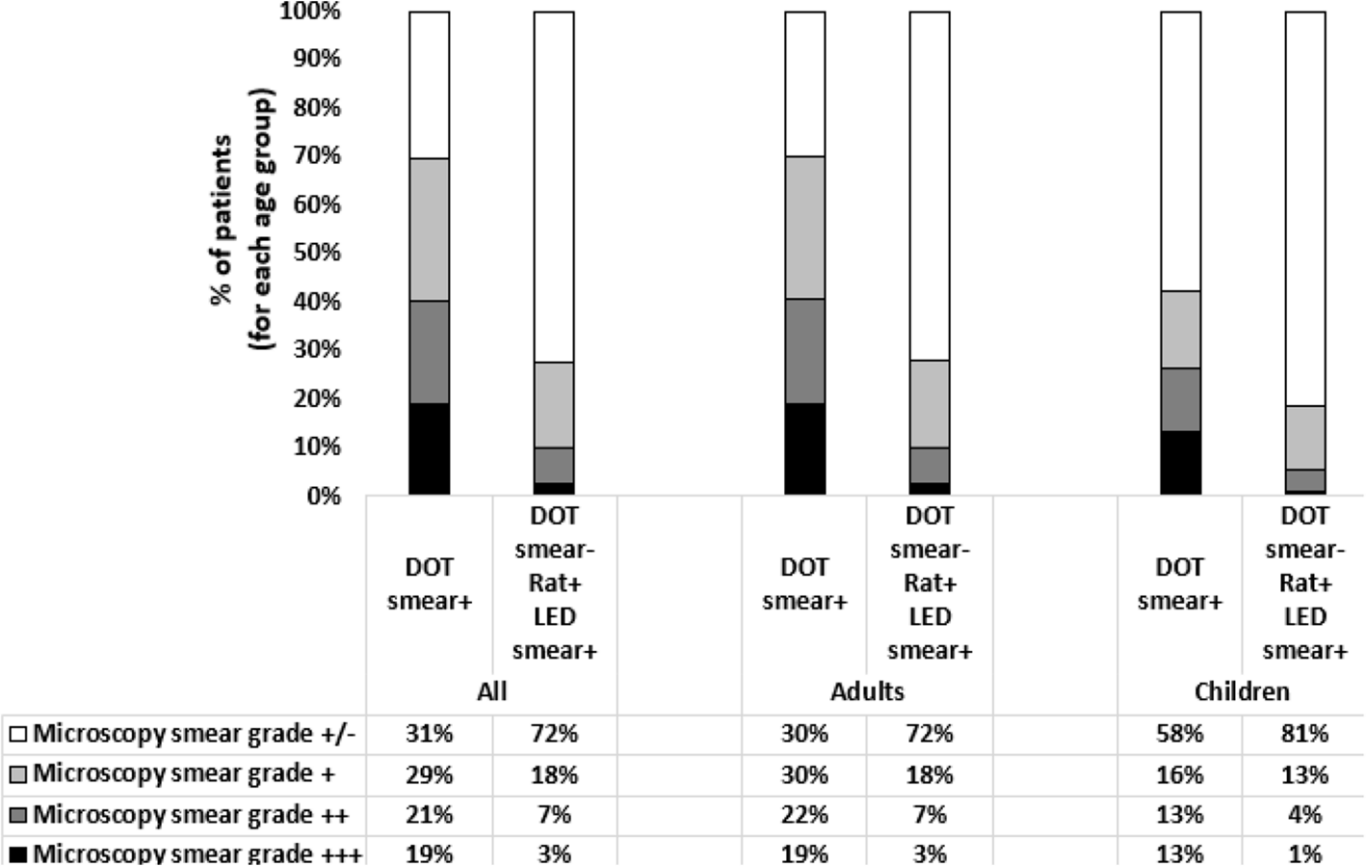
Tuberculosis cases detected by smear microscopy at DOTs and by rats at APOPO and confirmed by LED smear microscopy in Tanzania study sites from January to December 2022

Of the 3929 TB cases, APOPO rats’ detection contributed 52% (2029/3929) of the TB cases reported as smear-positive TB at DOT facilities that otherwise would have been missed by routine program. Compared to DOT facilities, the detection rats were six-fold more likely to detect TB among patients with Acid Fast Bacilli (AFB) smear 1+ or scanty [90% (1829/2029) versus 60% (1139/1900), odds ratio, OR = 6.11, 95% confidence interval, CI: 5.14–7.26]. The odds of identifying TB cases by rat among children were two-fold higher than adults [71% (91/129) versus 51% (1795/3542), OR = 2.3, 95% CI: 1.59–3.42] (Table 1). Furthermore, the odds of identifying TB cases by rats among children with AFB 1+ or scanty smear were two-fold higher than those among adults with the same MTB-load range [5% (86/1703) versus 3% (28/1067), OR = 2.0, 95% CI: 1.28–3.03] (Tables 2 and 3 and Figs. 3 and 4).

**Table 2:** Tuberculosis case detection in Tanzania using rats at APOPO and sputum-smear microscopy at DOT facilities by age and *Mycobacterium* bacillary load

	TB by detection rats at APOPO lab			TB by smear at DOT lab		
		Children	Adult		children	Adult
Bacillary load (AFB^a^﻿)	**N**	**n (%)**	**n (%)**	**N**	**n (%)**	**n (%)**
scanty	1364	74 (5)	1290 (95)	545	22 (4)	523 (96)
1+	339	12 (4)	327 (96)	522	6 (1)	516 (99)
2+	136	4 (3)	132 (97)	382	5 (1)	377 (99)
3+	47	1 (2)	46 (98)	336	5 (1)	331 (99)
Total	1886	91 (5)	1795 (95)	1785	38 (2)	1747 (98)

^a^Acid Fast Bacilli (AFB)

*APOPO* Anti-Persoonsmijnen Ontmijnende Product Ontwikkeling

*DOT* Directly Observed Therapy

**Table 3:** Tuberculosis patients in Tanzania evaluated using rats at APOPO by *Mycobacterium* bacillary load between children and adults

		Children	Adult			
Characteristic^a^	N	n (%)	n (%)	OR	95% CI	***p-***value
1+ or Scanty by detection rat	1703	86 (5)	1617 (95)	2.0	1.28–3.03	0.002
1+ or Scanty by smear at DOT	1067	28 (3)	1039 (97)			
**Total**	**2770**	**114**	**2656**			

^a^Acid Fast Bacilli (AFB)

*APOPO* Anti-Persoonsmijnen Ontmijnende Product Ontwikkeling

### Tuberculosis detection by smear at DOTs and by rats at APOPO confirmed by LED smear by *Mycobacterium tuberculosis* bacillary load

Higher proportions of AFB Scanty were identified among TB diagnosed by rats and confirmed by LED smear than those diagnosed at DOT, and this was similar among children and adults (Fig. 4).

**Fig. 5:**
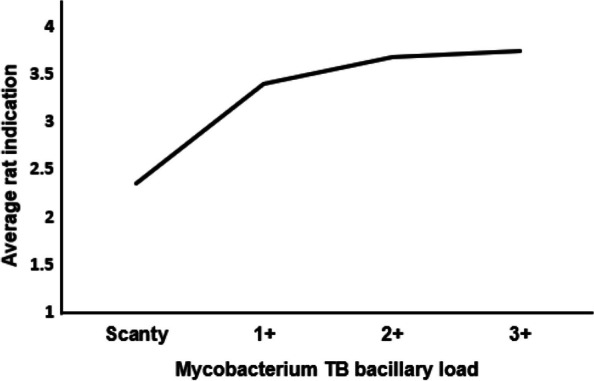
Average rat indication by *Mycobacterium Tuberculosis* bacillary load

On average, 2.36 (3459/1466), 3.40 (1233/363), 3.68 (545/148), and 3.75 (195/52) rats from a set of five rats indicated TB in those with AFB Scanty, 1+, 2+, and 3+, respectively More rats were able to identify TB as the MTB-load increased (Fig. 5).

## Discussions

For more than a decade, APOPO-trained African giant pouched rats have been used to re-evaluate (second-line screening) sputum samples tested by smear under routine program conditions [3, 4], and this has allowed us to compare the effect of rats on pulmonary TB case finding against DOT facilities. In the present study, rats demonstrated a 52% contribution in the detection of TB cases among patients who presented to routine DOT facilities with symptoms of TB and were diagnosed with TB. Rats were six times more likely than DOT facilities to uncover TB in patients with AFB smear 1+ or scanty, and this finding is similar to a previous study (five times more likely), though the latter was only applicable to children [4]. It is worth noting that if verification tests following the sputum samples’ rat indication as positive had been confirmed by molecular WHO-recommended rapid diagnostics, such as Xpert MTB/RIF Untra or Truenat, or by culture, which can detect TB with much lower concentrations of mycobacterium in a sample than concentrated LED microscopy, the contribution of rats would have been higher. Rats’ contribution has implications for Tanzania’s efforts to eliminate TB, especially as those who were identified using rats were deemed TB negative (missed TB cases) in the context of the standard TB diagnostic procedures and would have been left untreated otherwise.

We described elsewhere how rats can discriminate *Mycobacterium* TB by sniffing a variety of VOCs [3, 5]. According to a recent meta-analysis (53,181 samples from 24,600 patients in seven studies), the sensitivity and specificity of TB screening using rats were 81.3 and 73.4%, respectively [16]. In our report, we further analyzed the rats’ responses to different MTB-loads with respect to their impact on children and adults with pulmonary TB.

### TB detection using rats among children and adults

Comparing the effect of TB detection using rats in children to that in adults, rats were more than twice as likely to identify TB in children compared to adults, even though the likelihood of TB diagnosis among children with presumptive TB was lower by 58%. It should be noted that the increased TB detection among children was not only higher, but it was also significantly higher among children with a lesser bacillary load (AFB 1+ or scanty) compared to adults with a similar bacillary load. Our finding suggests that the rat-based technology could be used to improve pediatric TB case detection in high-burden countries such as Tanzania, where children are diagnosed with TB less frequently than adults by the standard of care.

In the previous study, rats contributed 39% (208/539) to pulmonary TB case detection reported in children (0–14 years), which was far less than our finding (71%, *p < 0.001*) [8].

APOPO’s rat technology has shown further improvement over time toward better rat and rat-handler training techniques, among other possibilities, which may explain the difference between the two reports. APOPO is working to advance its technologies further in this direction, and rats’ performance-rewarding systems are being computerized. The recent fully automated (i.e., controlling indication threshold time, notification of correct identification, reward delivery, and data recording are all computer controlled) rat cage is one step in this process and is currently being evaluated [14]. If validated, the automated system eliminates the effect of the human factor, i.e., rat handlers, who previously rewarded rats manually by providing food after a rat indicated the sample had TB. With the automated cage, APOPO anticipates improving and speeding up TB detection in Tanzania. In agreement with Mgode et al., our work has implications for developing rat-based TB diagnostic techniques or algorithms among children due to the challenges of detecting TB in this age group, where sputum quality and quantity remain a concern [7, 8].

### Fewer number of rats is needed to identify TB with a higher bacillary load

The direct relationship between smear positivity and MTB-load was expected [17]. Interestingly, we also observed that when the bacillary load increased, more and more rats identified TB cases, suggesting that fewer rats were needed to detect TB, and once again, the effect tends to be stronger in children than in adults. The properties of the chemical signature, VOCs, among various age groups have not yet been determined, and the previous report was for adults only [5]. Our research led us to two hypotheses: (1) children may have distinct or greater VOC characteristics, making it simpler to diagnose TB even when the bacillary load is lower than that of adults; and (2) probably, the less developed physiology may not let them to produce other odors that could be developed by adult patients, which in turn may somehow mask the odor from the characteristic VOCs. These merit additional investigation. On the other hand, sputum quality and quantity have an impact on the accuracy of TB diagnosis by smear or Xpert MTB/RIF (Cepheid, Sunnyvale, CA, USA) [17–19], and in the same vein, higher-quality sputum makes it simpler for rats to detect TB.

Our study has some limitations. First, because the data were from a routine program setting, the age of certain TB cases was missing, which prevented them from being further analyzed. Rats were, however, four times more likely to detect TB than DOT among those with missing ages, and It’s likely that the conclusion would remain unchanged if the missing data were included. Second, health workers at various DOT facilities may not have received training before the data collection, and the TB screening and recording procedures may not have been consistent, which may result in inconsistent presumptive patient identification. Third, despite the fact that sputum induction is usual at DOT facilities, it was possible to collect insufficient sputum samples from children, which may have resulted in fewer pediatric TB diagnoses than in adult patients. Finally, for those samples evaluated by rats after the Xpert MTB/RIF negative test result, using the same sample for rats was not possible. Thus, a sister sample was collected and evaluated by rats. In such cases, we cannot rule out the possibility of inter-sample variations, i.e., the sister sample had a chance not to be negative if it was tested by Xpert MTB/RIF before rats’ evaluation.

In conclusion, in our high TB burden settings, APOPO rats led to more than half of the TB cases identified. Children had a higher rate of TB detection, particularly among those with lower bacillary loads. In the present study, rats demonstrated their essential role in TB control efforts, specifically in identifying TB cases that were overlooked in routine program diagnostic settings. Since rats were more easily able to detect TB in children than adults, and the data from prior reports about chemical signatures, VOCs, were only available for adults, further research describing the characteristics of VOCs in children versus adults may pave the way to enhance TB diagnosis in children. Like other TB diagnostic technologies, it’s likely that the quality of the sputum specimen influences the rat’s ability to detect TB by sniffing; therefore, advancements in this area make it simpler for the rats to recognize TB.

## Data Availability

The authors confirm that, for approved reasons, some access restrictions apply to the data underlying the findings. Although the patient-level data do not include patient names, this IRB decision is in the interest of ensuring patient confidentiality. An individual may email the lead author (tefersast@gmail.com) or the APOPO TB Detection Research Department (tefera.agizew@apopo.org) to request the data.

## Abbreviations

AFB: Acid fast bacilli
APOPO: Anti-persoonsmijnen ontmijnende product ontwikkeling
CI: Confidence interval
CRF: Case report form
DOT: Directly observed therapy
LED-FM: Light-emitting diode fluorescence smear microscopy
MTB: *Mycobacterium Tuberculosis*
OR: Odds ratio
RIF: Rifampicin
STATA: South Texas Art Therapy Association
TB: Tuberculosis
VOC: Volatile organic compound
ZN: Ziehl-Neelsen
